# Alterations in MicroRNA gene expression profile in liver transplant patients with hepatocellular carcinoma

**DOI:** 10.1186/s12876-020-01596-2

**Published:** 2021-06-12

**Authors:** Afsoon Afshari, Ramin Yaghobi, Mohammad Hossein Karimi, Javad Mowla

**Affiliations:** 1 Shiraz Nephro-Urology Research Center, Shiraz University of Medical Sciences, Shiraz, Iran; 2 Shiraz Transplant Research Center, Shiraz University of Medical Sciences, Shiraz, Iran; 3 Genetic Department of Tarbiat, Modares University, Tehran, Iran

**Keywords:** LNA-array, microRNA, Liver, Transplantation, Hepatocellular carcinoma, RT-PCR

## Abstract

**Background:**

Hepatocellular carcinoma (HCC) can lead to liver failure which renders to liver transplant. miRNAs might be detected as biomarkers in subclinical stage of several hepatobiliary disorders like HCC. Therefore, in the present study, alterations in miRNAs as biomarkers were detected in LT patients with HCC.

**Methods:**

Fourteen tissue samples composed of 5 rejected and 9 non-rejected ones were used for studying the miRNAs expression pattern using LNA-array probe assay and the result was evaluated by in house SYBR Green Real-time PCR protocols on 30 other tissue samples composed of 10 rejected and 20 non-rejected ones for the selected miRNAs. All samples were collected from liver transplanted patients with HCC.

**Results:**

The study results revealed that in rejected patients compared to non-rejected ones, hsa-miR-3158-5p, -4449, -4511, and -4633-5p were up-regulated and hsa-miR-122-3p, -194-5p, 548as-3p, and -4284 were down-regulated. ROC curve analysis also confirmed that miR194-5p and -548as-3p in up-regulated and also, miR-3158-5p, -4449 in down-regulated microRNAs are significantly important molecules in rejection.

**Conclusion:**

Finally, the tissue levels of specific miRNAs (especially hsa-miR-3158-5p, -4449, -194-5p and -548as-3p) significantly correlated with the development of HCC, which can be present as biomarkers after further completing studies.

**Supplementary information:**

The online version contains supplementary material available at 10.1186/s12876-020-01596-2.

## Background

The main treatment approach in many end stage liver failures is liver transplant (LT) [1]. Ranked as the fifth most common diagnosed cancer worldwide, hepatocellular carcinoma (HCC) refers to a multistage process, involving many genes [2], which can render to hepatocyte turnover, oxidative DNA damage, and inflammation [3]. Even though innovated therapeutic strategies are being established, handling advanced HCC is poorly efficacious and merely at the point of diagnosis. Apparently, transplantation seems to be the only known effective treatment approach for this disease [4].

A considerable part of human genome is consisted of non-coding regions, including non-coding RNAs, known as microRNAs (miRNAs) [1]. These regions encode dynamic miRNAs, regulating around 20–30% of mammalian genes that comprise nearly 5% of transcriptome [1, 5]. MiRNAs are evolutionary conserved, made up of 18–25 nucleotides. They are also involved in various biological processes e.g. cell growth, apoptosis, hematopoietic lineage differentiation, and gene regulation [1, 6, 7].

There are also many miRNAs with specific expression patterns in the liver at different stages [8]. Investigations have demonstrated that some miRNAs are involved in regulating many metabolic pathways. Changes in miRNA expression levels might reflect upon the underlying sources of inflammation [9]. The research which was conducted by Murakami et al., in 2006 was one of the pioneer studies on investigation the role of miRNAs in HCC development [10]. After that a number of studies have been investigated the miRNA changes in HCC [11–15]. As an illustration, it has been reported that miR-17-92, miR-21, miR-221, miR-222, and miR-224 are frequently up-regulated in HCC. Furthermore, the importance of miRNAs in liver function has been broadly documented in the related literature [16]. Studies have also revealed that miR-122 as a blood biomarker changes in viral, alcoholic, and chemical injuries induced in the liver [8]. The importance of miRNAs is also, studied in LT rejection and it seems that some of miRNAs are playing specific role in this regard. Ectopic expression of serum miRNA signatures is known to have prognostic, diagnostic, and biologic impact in liver allograft rejection [17]. Other sources showed that in rat model of LT rejection, miR-146a, 15b, 223, 23a, 27a, 34a and 451 were upregulated [18].

Furthermore, different studies have been used the LNA-array technique for detection of miRNAs’ level in different samples and discussed the advantages of this method over other related methods. The ability of this method to produce high affinity hybridizations render to accurate discriminating signals, have been announced in earlier reports [5, 19–21].

Based on invasiveness and time-consuming nature of the pathological tests, mainly biopsy, the necessity for a faster and less invasive method seems crucial. Therefore, LNA-array (locked nucleic acid-array) probe and real-time polymerase chain reaction (RT-PCR) was utilized to detect alterations in miRNA expression in the tissues of LT patients with HCC. This study is tried to elucidate the role of miRNAs in regulating the rejection of liver transplant in HCC patients.

## Methods

### Patients and samples

This study was fulfilled in the Pathology Ward of Namazi Hospital, Shiraz, Iran (2016–2018). To conduct this study, a total number of 44 formalin-fixed paraffin-embedded (FFPE) samples were collected from LT patients with HCC. Macro-dissection was also performed. In brief, there were attempts to adopt a standard procedure to dissect the middle region of the tumor to harvest a proper sample, surrounded by the cancerous cells. The harvesting procedure was then confirmed microscopically to ensure the nonexistence of non-cancerous cells in each sample. All of the participants were asked to completed a questionnaire on demographic characteristics, history of other cancers except HCC, alcohol use, and tobacco use. Those with a history of other cancers, alcohol use, or tobacco use were excluded from the study.

As this study was a retrospective one, the samples were collected and divided into acute rejected and nonrejected patients due to their pathology reports. The pathology results were closely examined by expert pathologists and all of tissue samples that were classified as rejected were at stages III and IV based on AJCC (American Joint Committee on Cancer) staging system [22].

**Fig. 1:**
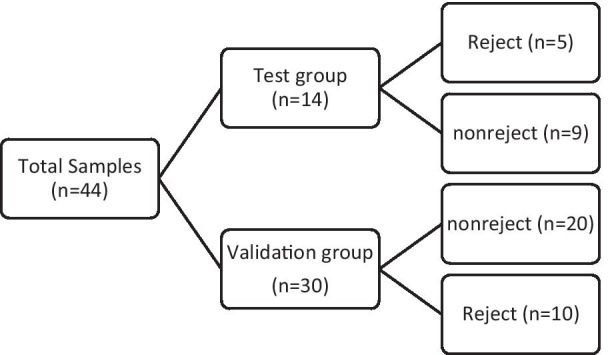
The study sample division chart. The studied patents samples were divided into two categories. The first category was composed of 14 individuals (test group) which were participated in LNA-array probe test and were composed of 5 rejected and 9 nonrejected samples. The second group was consisted of 30 individuals (validation group). These patient samples were used for validating the results of LNA-array test

The samples were divided into two main groups. The 1st group (test group) was composed of 14 liver transplant samples (5 rejected and 9 nonrejected). The 2nd group (validation group) was composed of 30 samples (10 rejected and 20 nonrejected). For more clarification the sample division is shown in Fig. 1. All the protocols applied in this study were in complete agreement with the Helsinki Declaration and its later amendment. The local Ethics Committee of Shiraz University of Medical Sciences also approved each stage of this study. After explaining the study objectives, written informed consent was further obtained from each patient.

### De-paraffinization of FFPE samples, RNA isolation and quality control

Using a microtome, 10 cuts of 5 μm of each sample were prepared. All the samples were RNA isolated, using Trizol™ (Invitrogen, Carlsbad, CA, USA) according to the manufacturer’s protocol. Sample RNA Quality Control was further performed by an Agilent 2100 Bioanalyzer, to provide an electropherogram for each sample. In addition to the measurement of the rRNA ratio in a traditional manner (28S/18S), the Bioanalyzer provided an RNA Integrity Number (RIN) value (ranging from 0 to 10) and a reliable impression of RNA quality. The recommended RIN values were higher than 7 for good array performance. The NanoDrop instrument was also used to measure the concentration (A260), protein contamination (ratio A260/A280), and contamination with buffer components, or organic compounds (ratio A260/A230) accurately.

### Sample preparation for LNA-array probe

#### Labeling, hybridization, and normalization

Using the miRCURY LNA™ miRNA Hi-Power Labeling Kit (Exiqon, Denmark), 400 ng total RNA were labeled by Hy3™ and Hy5™ fluorescent label for labeling the samples and the references, respectively to produce highly efficient and uniform labeling. The samples (labeled with Hy3™) and the references (labeled with Hy5™) were then mixed pairwise and recruited for hybridization. The hybridization procedure was performed using Tecan’s HS 4800™ hybridization station (Tecan, Austria) followed by miRCURY LNA™ miRNA array instruction manual.

Later, the labeled specimens were hybridized by miRCURY LNA™ miRNA array 7^th^ Gen (Exiqon, Denmark**)** using the manufacturer’s protocol. The quantified signals of the background were also corrected (Normexp with offset value 10) and normalized using the global Locally Weighted Scatterplot Smoothing (LOWESS) regression algorithm.

### Principle component analysis (PCA) plot

In order to decrease the proportions of the bulky data and to explore sample classes arising naturally and according to the expression profile, the PCA plot was applied. The top 50 miRNAs that had major variations across all the samples were also included.

### Array slide quality control using spike-ins (labeling controls) and normalization

The spike-in controls were added in various concentrations in both Hy3™ and Hy5™ labeling reactions, giving the opportunity to evaluate the labeling reaction, hybridization, and performance of array experiment in a general manner (The level correlation of the signal intensities across all the slides is shown in the Additional file 1: Figure A1).

The LOWESS also allows the correction of systematic deviations in the MA plot resulting in an intensity-dependent adjustment of the MA-data to a straight line. The positive effect of this normalization is illustrated in the MA plots for each slide, showing the plot before and after normalization (see Additional file 1: Figure A2).

### Real-time PCR

#### Poly A polymerization, cDNA synthesis, and real-time PCR

To this end, for study the expression level of selected miRNAs in the validation group of samples, 5 µg of each isolated total RNAs was used for poly A polymerization by means of microRNA cDNA Synthesis Kit (Parsgenome, Iran). Then, 2 µg of the product was applied for the next step, i.e., cDNA synthesis using specific reverse primers for each miRNA. Finally, these miR specific cDNAs were employed as templates for quantitative SYBR Green Real-time analysis (Parsgenome, Iran). The expression level of the selected miRNA molecules was further determined employing SYBR Green Real-time PCR (ABI, USA). In addition, U6 miRNA primers were included in the mentioned kit as an internal control.

The mix used for Real-time PCR for each reaction contained SYBR Green Premix (10 µl of Ex taq, Takara, Japan), Rox reference dye (0.4 µl), forward and reverse primers (1 µl of 10 pM mix of each primer pairs), and template (2 µl of synthesized cDNA, between 0.1 to 100 ng). Nuclease free water also added to this reaction up to 20 µl total volume. The used forward primer sequences are shown in Table 1. The program employed for Real-time PCR was one cycle 95 °C -5 min, followed by 40 cycles of 95 °C- 5 s, 61 °C- 20 s, and 72 °C- 30 s, followed by melting curve analysis for specificity of each reaction.

**Table 1:** The forward primer sequences used for validating the selected microRNAs

miRNA name	Sequence (5′ to 3′)
has-miR122-3p	AACGCCATTATCACACTAAA
has-miR4284	GGGCTCACATCACCCCAT
has-miR194-5p	TGTAACAGCAACTCCATGTGG
has-miR548as-3p	TAAAACCCACAATTATGTTTG
has-miR4511	GAAGAACTGTTGCATTTGCC
has-miR4633-5p	ATATGCCTGGCTAGCTCCT
has-miR4449	CGTCCCGGGGCTGCGCGA
has-miR3158-5p	CCTGCAGAGAGGAAGCCC

### Statistical analysis

The data were coded and imported into the IBM SPSS Statistics software (version 24). The quantitative variables were then compared using the non-parametric Kruskal–Wallis test for a comparison between more than two groups and the Mann–Whitney U test was employed to compare two groups. The calculations for expression comparison in LNA-array tested samples were also executed by the R/Bioconductor software, using the LIMMA package. The fold changes in the validation group of samples were calculated by livak method (2^−ΔΔct^). The *P*-value cut-off score for a significant difference was considered less than 0.05.

## Results

### Descriptive statistics

The test group was composed of 14 LT patients with the mean age of 47.4 ± 7.8 years (age range 31–56) and included 9 (64.3%) males. These samples were used for LNA-array probe. More data is shown in Fig. 1 and Table 2. The validation group consisted of 20 LT patients with no acute rejection of LT and 10 patients showing acute rejection. The mean age of nonrejected ones was 49 ± 15.3 years (age range 14–69) and included 14 (70%) males. The rejected samples had mean age of 49.7 ± 17.1 years (age range 13–68) and included 6 (75%) males.

**Table 2:** Descriptive statistics related to first group (test group) of patients; the total number of patients participating in the test group and the number and percent of male and female participated in test group is shown in the table

			Rejection condition		Total
			Rejected	Nonrejected	
Gender	Male	Count	3	6	9
		% within gender	33.3%	66.7%	100.0%
		% within rejection	60.0%	66.7%	64.3%
	Female	Count	2	3	5
		% within gender	40.0%	60.0%	100.0%
		% within rejection	40.0%	33.3%	35.7%
Total		Count	5	9	14
		% within gender	35.7%	64.3%	100.0%
		% within rejection	100.0%	100.0%	100.0%

### PCA plot analysis

**Fig. 2:**
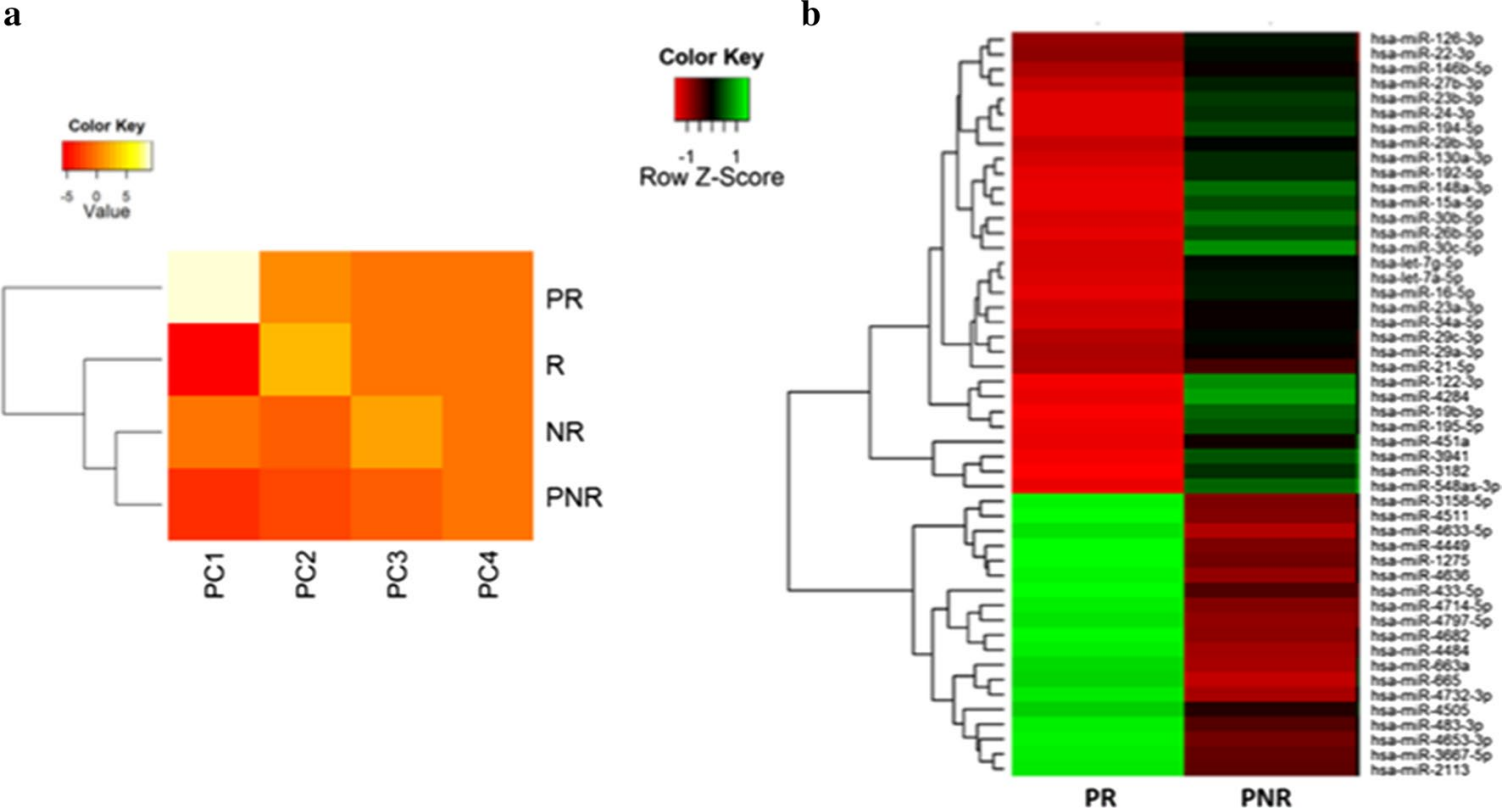
(**a**) Matrix PCA plot. The PC1 to PC4 analysis was performed on the results of microRNA expression variation using LNA-array probe on test group of samples; due to the color key information, the most differences belong to PC1 and between samples PR and PNR. Normalized log ratio values were used for the analysis. (**b**) Each row represents a microRNA and each column represents a sample. The color scale illustrates the relative expression level of microRNAs. Red color represents an expression level below the reference, and green color represents expression higher than the reference genes. Pooled sample groups were used in order to reach more exact analyses and the results mentioned here is related to pooled samples. Here, PR means pool of reject samples while PNR means pool of nonrejected samples and R means one reject sample while NR means one nonrejected sample

The top 50 miRNAs that had major variations across all the samples were used for PCA plot analysis. The results are depicted in Fig. 2a, demonstrating variations of the biological patterns or technical factors in the test group (n = 14). Accordingly, PC1 and PC2 describe variations related to sample groups or treatments, whereas PC3 and later PC’s might describe underlying or less variable factors like sample preparation conditions, operator, storage time, etc.

The unsupervised PCA and the hierarchical clustering could clearly show a tight grouping of the control samples (i.e., the non-rejected), whereas the treated samples (namely, the rejected) differed both from the controls as well as from each other. A simple expression analysis by comparison did detect a subset of differentially expressed miRNAs including four up-regulated and four down-regulated miRNAs selected for further analyses.

### Heat map and unsupervised hierarchical clustering

The heat map diagram shows the results of a two-way hierarchical clustering of miRNAs and the samples in the test group (n = 14). The clustering was done using the complete-linkage method together with the Euclidean distance. A small subset of the miRNAs was also excluded from the heat map. As well, the normalized log ratio values were used for the analysis (Fig. 2b).

### MiRNA expression analysis for rejected versus non-rejected in LNA-arrayed samples

An expression analysis for the rejected cases versus the non-rejected ones was performed using a simple comparison method in the test group (n = 14). The miRNA profiling accordingly identified a subset of top miRNAs (where the absolute value of the log fold change was larger than 1) out of the total number of miRNAs analyzed (371) by the miRCURY LNA™ miRNA Array, differentially expressed in the rejected and the non-rejected samples. Table 3 provides a list of miRNAs with the most differential expression. The results showed that hsa-miR-122-3p, -548as-3p, -4284, and -194-5p had the highest levels of reduction amongst all the studied miRs, and hsa-miR-4511, -4449, -4633-5p, and -3158-5p were the most increased ones; respectively.

**Table 3:** The table shows the selected differentially expressed microRNA candidates, ranked according to the absolute value of the log fold change from the test group of samples. Also, the table shows the average Hy3 (fluorescent dye) used for detecting the expression level (number of microRNAs tested) and the fold change of each microRNA in each category (nonrejected and rejected). Finally, logFC (logarithmic fold change) of each microRNA calculated in order to understand the extent of expression of each microRNA in reject samples in comparison to nonrejected group

Annotation	Average Hy3	Nonreject fold change	Reject fold change	Log FC
hsa-miR-122-3p	9.025	1.068	− 1.013	− 0.793
hsa-miR-194-5p	9.161	0.606	− 1.051	− 0.258
hsa-miR-548as-3p	8.661	0.015	− 1.714	− 1.326
hsa-miR-4284	13.779	0.893	− 1.069	− 0.759
hsa-miR-3158-5p	8.303	− 1.651	0.745	0.873
hsa-miR-4449	7.259	− 1.399	0.935	1.035
hsa-miR-4511	7.337	− 1.656	0.775	1.204
hsa-miR-4633-5p	8.176	− 1.704	0.658	0.973

### Selected miRNA expression level comparison between rejected and non-rejected groups of patients after LT

**Fig. 3:**
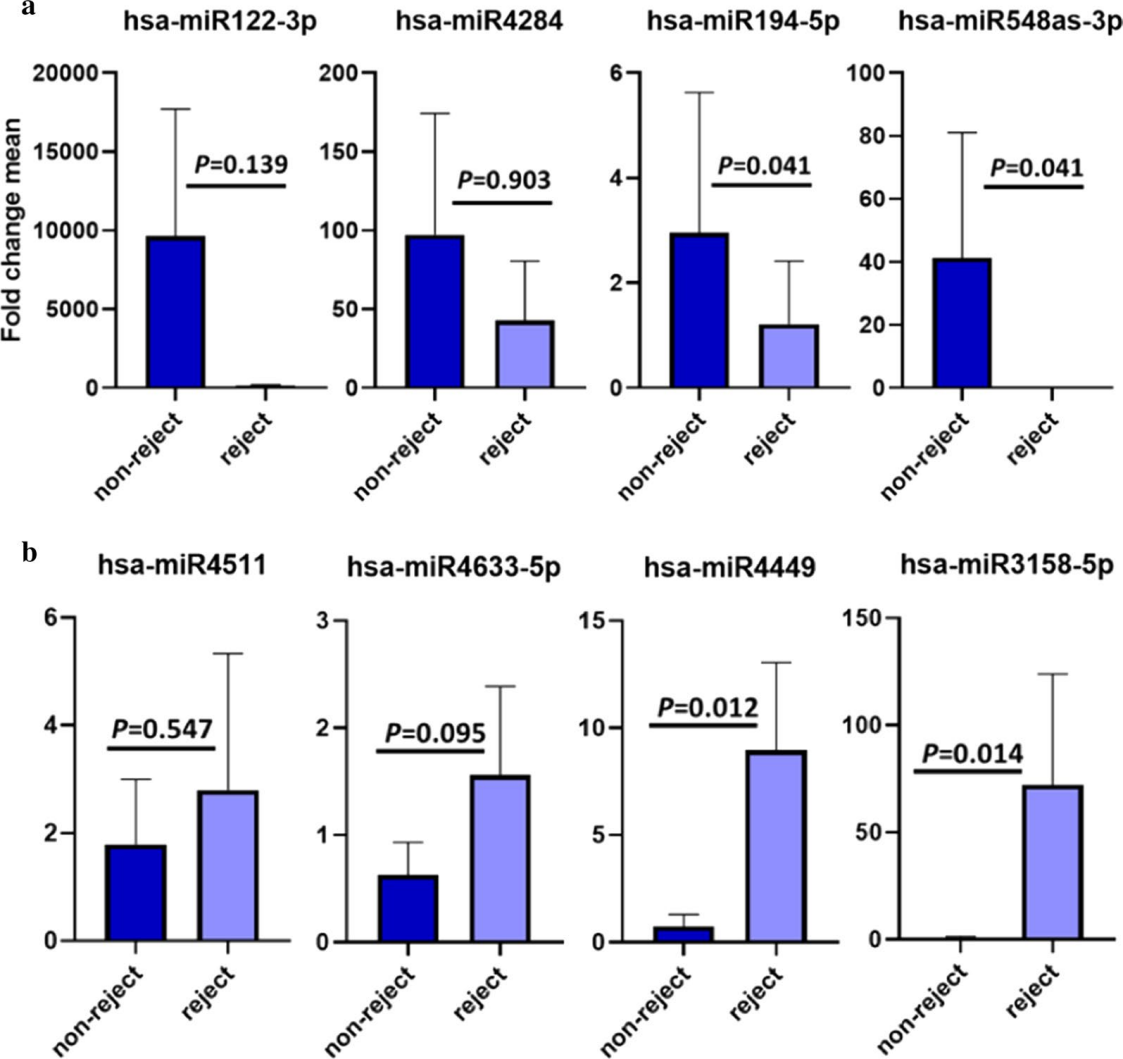
The expression level of selected miRs for down regulated miRs (**a**) and for up regulated ones (**b**) based on fold change. The results of LNA-array probe test on the test group of samples were further validated using qRt-PCR on the validation group of samples. The selected microRNAs had the most variation among all the tested microRNAs. The fold changes are calculated by livak method (2^−ΔΔct^)

The expression level of the selected miRs was tested in the 2nd groups of patients (validation group) composed of rejected and non-rejected ones. The results of this selection are demonstrated in Fig. 3a for the down-regulated miRs and Fig. 3b for the up-regulated ones based on a fold change. The results also revealed that hsa-miR-548as-3p and -194-5p had significantly reduced and hsa-miR-4449 and -3158-5p had shown a significant rise in the patients with LT rejection.

### Calculating sensitivity and specificity of down-regulated miRNAs between nonrejected and rejected groups of patients after LT

**Fig. 4:**
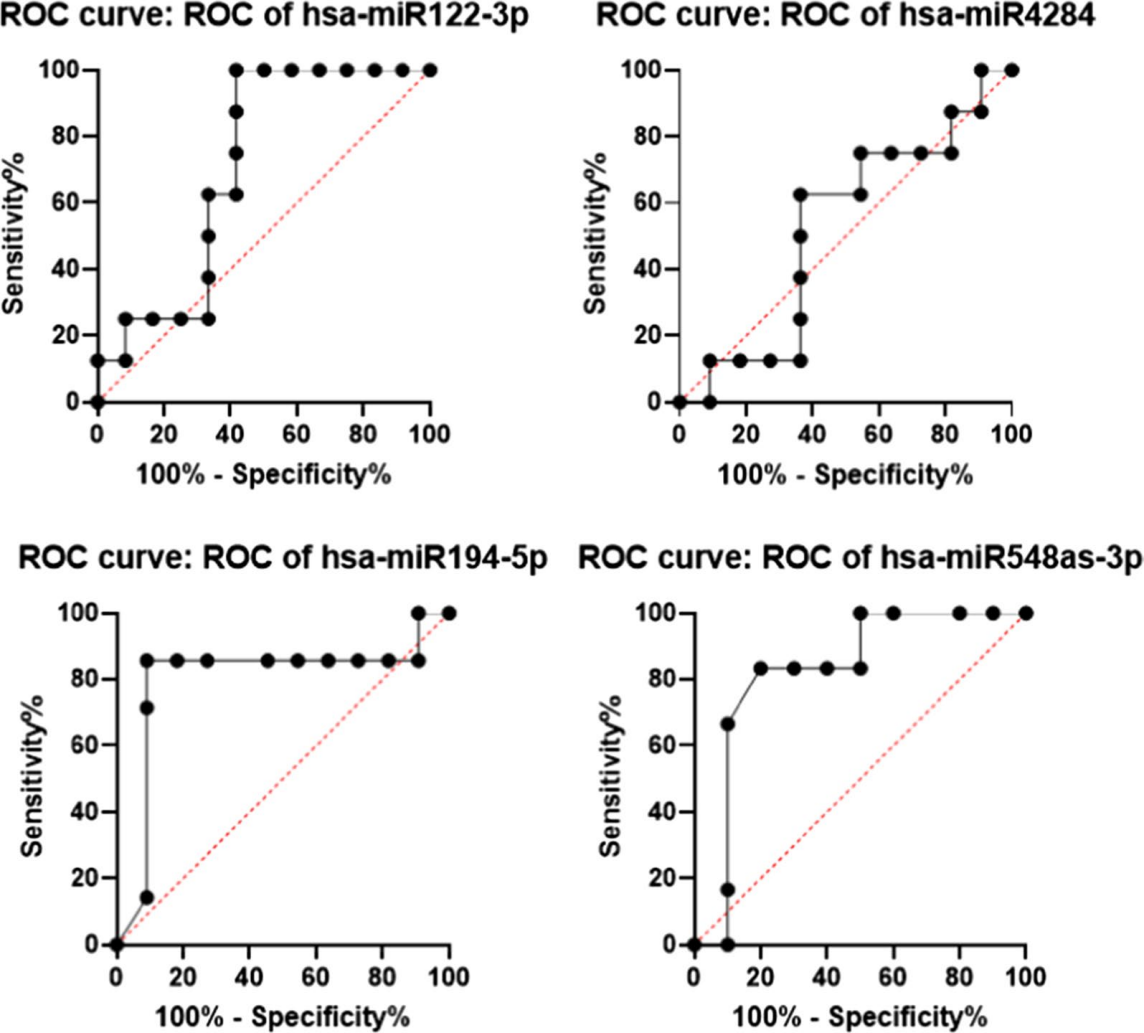
Calculating the sensitivity and specificity of down-regulated microRNAs between nonrejected and rejected groups of patients after transplantation. In order to further validate the sensitivity and specificity of selected down-regulated microRNAs between nonrejected and rejected groups of patients, the ROC curve analysis performed

The sensitivity and the specificity of the down-regulated miRNAs between the non-rejected and rejected samples of patients in the validation group were calculated (Fig. 4). The area under curve (AUC), *p* value, and 95% confidence interval (95% CI) of each down-regulated miRNA are demonstrated in Table 4. Two of the miRNAs (i.e., has-miR194-5p and -548as-3p) had a significant difference (*p* < 0.05), making them candidates as promising biomarkers.

**Table 4:** Sensitivity and specificity of down and up-regulated microRNAs between nonrejected and rejected groups of patients (validation group) after transplantation. The AUC and *p* value of each microRNA is shown to describe the importance of each studied microRNA

miRNA	Area under the curve (AUC)	*p* value	95% CI	Down or up-regulated
has-miR122-3p	0.7083	0.1228	0.4740–0.9427	Down-regulated
has-miR4284	0.5228	0.8688	0.2506–0.7949	Down-regulated
has-miR194-5p	0.7987	0.0372	0.5414–1.000	Down-regulated
has-miR548as-3p	0.8250	0.0344	0.645–1.000	Down-regulated
has-miR4511	0.6000	0.5152	0.3134–0.8866	Up-regulated
has-miR4633-5p	0.7424	0.1078	0.4985–0.9864	Up-regulated
has-miR4449	0.8385	0.0122	0.6620–1.000	up-regulated
has-miR3158-5p	0.8561	0.0182	0.5946–1.000	Up-regulated

### Calculating sensitivity and specificity of up-regulated miRNAs between nonrejected and rejected groups of patients after LT

**Fig. 5:**
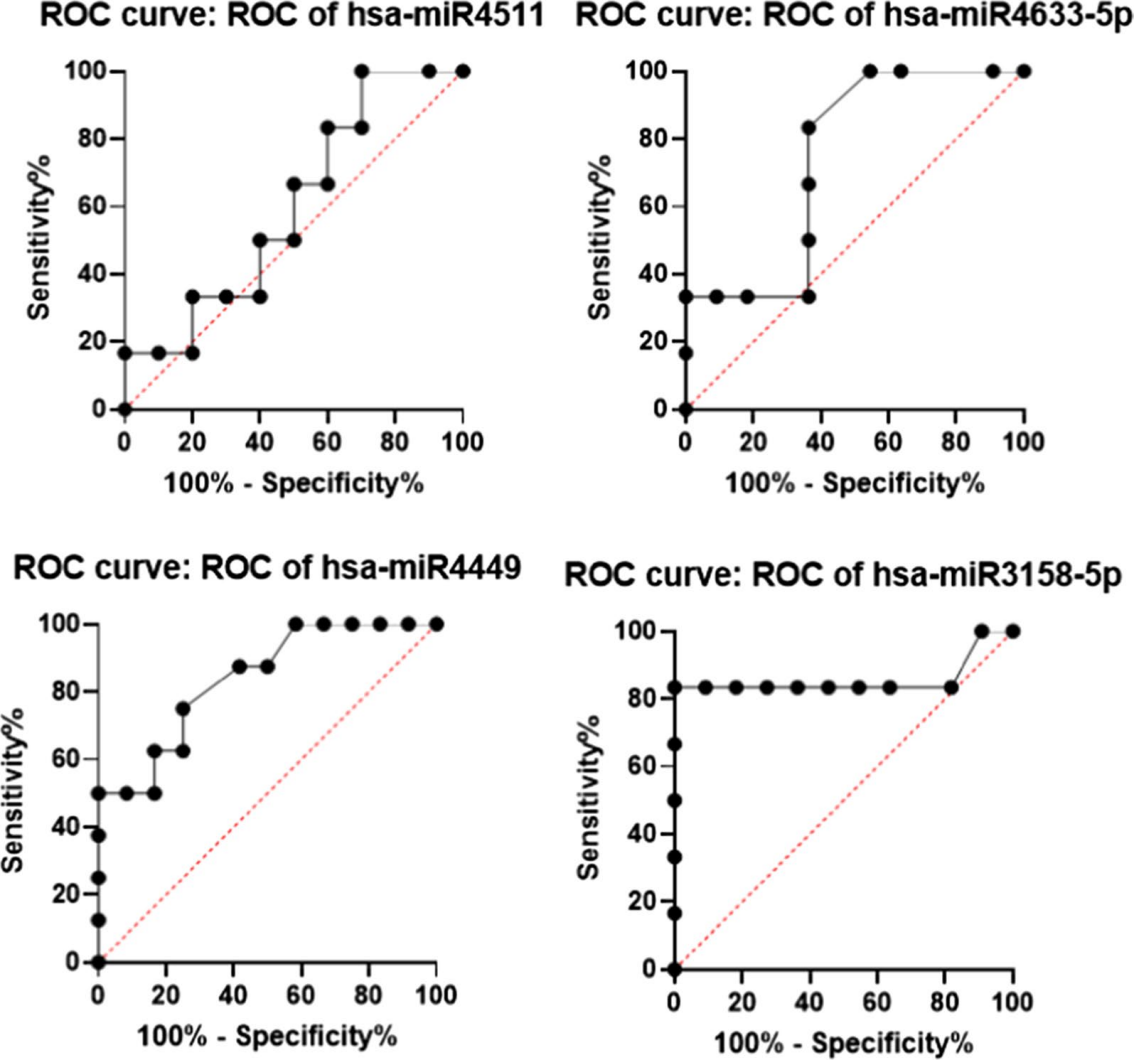
Calculating the sensitivity and specificity of up-regulated microRNAs between nonrejected and rejected groups of patients after transplantation. In order to further validate the sensitivity and specificity of selected up-regulated microRNAs between nonrejected and rejected groups of patients, the ROC curve analysis performed

The sensitivity and the specificity of the up-regulated miRNAs between the non-rejected and rejected samples of patients in the validation group were calculated (Fig. 5). The AUC, *p*-value, and 95% CI of each up-regulated miRNA are demonstrated in Table 4. Two of the miRNAs (namely, has-miR4449 and -3158-5p) established a significant difference (*p* < 0.05), making them candidates as promising biomarkers.

## Discussion

The functions of miRNAs can provide valuable diagnostic measurements as well as prognostic indicators for several diseases including types of cancer [5]. A healthy liver consists of different cells producing a variety of miRNA expression profiles according to complex intrinsic and extrinsic signals. Under microbial pathogenesis, these cells can also alter the profile of miRNAs [23]. Commonly, HCC is accompanied by loss of liver and the remaining treatment would be transplantation. Although many studied have been accomplished in order to find early detecting markers on the basis of miRNAs, still no effective biomarkers have been introduced in this regard. Knowing the fact that transplantation is the final therapeutic choice for HCC, the maintenance of the grafted liver and prevention from rejection is the priority for these patients. In the present research, it is tried to evaluate the alterations in miRNA expression profile of LT patients with HCC in order to introduce probable miRNA biomarker in early detection of rejection. Although it has been suggested that some of the circulating blood miRNAs have the potential to be used as miRNA-based blood biomarkers in cancer detection [24, 25], it is noteworthy to investigate the possibility of introducing more accurate miRNAs for following rejection outcomes. In a study done by Miyaaki et al., they used the same techniques as we did, for studying the miRNA profile of liver transplanted patients with HCV/HIV co-infection and confirmed that miR-101b and miR-149 are significantly decreased in co-infected group of patients comparing to HCV-infected ones [26].

The findings revealed that miR-4449, miR-4511, miR-3158-5p, and miR-4633-5p had been up-regulated and miR-4284, miR-122-3p, miR-194-5p, and miR-548as-3p down-regulated in patients with LT rejection once compared with non-rejected ones. The results from LT patients were also in accordance with the LNA-array probe results. With reference to the results collected from the PCA plot, there was a significant difference between miRNA expression in the rejected LT patients versus the non-rejected ones.

The miR-122-3p is a liver-specific miRNA, highly expressed, comprised of more than 70% of all cloned liver miRNAs, unlike other tissues [1, 27]. Moreover, this miRNA plays a role in the liver metabolism, for instance, many genes interfering in lipid metabolism regulation can be down-regulated when using an antisense strategy to knock it down [28]. The sequence and the expression pattern of the miR-122-3p in the liver is also highly conserved [29]. In a study, HCC patients had shown a reduction in miR-122-3p expression [30]. MiR-122-3p, miR-26a, and miR-195 have been also detected as tumor suppressors molecules in the liver [31]. The fact that miR-122-3p reduces in HCC patients was accordingly certified in the present study. Other studies on liver transplant recipients with recurrent HCV and acute cellular rejection demonstrated the importance of miR-122 and miR-194 in liver transplant rejection either [24, 32]. Furthermore, the correlation between miR122-3p and -194-5p has been a subject of research studies. Therefore, this miRNA was analyzed between HCC non-rejected and rejected patients, indicating that the given miRNA could significantly (*p* = 0.041) decrease in the rejected LT cases.

The miR-194-5p expression had been previously detected, reporting that such an miRNA could play a role in activation of stellate cells during liver fibrogenesis [33, 34]. Another study had further profiled the expression of miR-194-5p as a marker of liver HCC metastasis by showing the overexpression of miR-194 in cancerous liver cell lines to down-regulate N-cadherin expression and to suppress migration, invasion, and metastasis [31]. An increase in the expression of miR-194-5p in HCV-infected hepatoma cells has been also well documented although the miR-194-5p expression level had significantly reduced in HCC patients [35]. In addition, Farid et al. had demonstrated that the expression levels of miR-122 could significantly correlate with miR-194 among HCC patients (*r* = 0.322, *p* = 0.007). They had also reported no significant differences between non-rejected and rejected groups of patients in the expression of miR-194-5p (*p* = 0.134). However, the difference in miR-122 expression level between two studied group was significant (*p* = 0.001) [36]. In addition, miR-194-5p had been down-regulated in the tissues involved in HCC and was able to reduce cell viability and proliferation by inducing G1 arrest and apoptosis in HCC cells [37].

Some miRNAs might be originated from repetitive elements like transposable elements (TEs) [38]. Hsa-mir-548as-3p is also a member of a large human gene family, derived from transposable elements named Made1 [39]. Approximately 69 members are found in almost all human chromosomes, especially chromosomes 6, 8, and X (30.43%). The predicted targets for hsa-miR-548as-3p are also functional molecules in many biological processes like MAPK signaling, cell cycle, p53 signaling pathway, colorectal cancer (CRC), non-small cell lung cancer, B cell receptor signaling pathway, transforming growth factor β (TGF-β) signaling pathway and renal cell carcinoma [40]. TGF-β signaling pathway is also important in post-transplant inflammation. Besides, it is considered as one of the main mediators and inducers of fibrosis. Therefore, miRNA down-regulation during the rejection period renders to overexpressed SMAD4, enhancing the signaling pathway of TGF-β [41]. In addition, it has been reported that Tg737 is a target gene for miR-548as-3p, whose down-regulation facilitates HCC cell proliferation in vivo and in vitro, followed by an increase in colony formation [42]. The present study showed that the given miRNA had significantly (*p* = 0.041) reduced in rejected LT patients with HCC.

The miRNA-4248 is located on chromosome 7, but there is not much data published about it. One study had used cross-mapping to detect miRNAs and had reported hsa-miR-4284 in cross-mapping events [43]. In addition, another research team had reported that hsa-miR-4284 could be the most down-regulated ones in the tissue samples of patients with ulcerative colitis. They had further claimed that miR-4284 level correlated with the disease activity by regulating CXCL5 mRNA expression ^44^. Another research had found that miR-4284 and -4484 could be considered as diagnostic biomarkers for diffuse large B-cell lymphoma (DLBL) [45]. The down-regulation of this miRNA was further detected in LT patients with HCC in the present study. Furthermore, the sensitivity and the specificity of analyses of the four down-regulated miRNAs showed that miR194-5p and miR548as-3p might have enough qualities (Table 4) for being biomarker candidates for rejection especially among HCC patients.

MiR-3158-5p is detected to be located on chromosome 10 and has more than 500 predicted targets. The bioinformatic studies here showed that *TGF-β2* gene could be observed among its upper 95% target scores (see Additional file 1: Table A1; “www.miRDB.org”). TGF-β also plays an important role in preparing a suitable microenvironment for tumor cell growth in liver diseases. This factor causes a signaling pathway that can promote progression of HCC in two ways; first, this factor has an intrinsic activity as an autocrine or paracrine growth factor, and second, it has extrinsic activity inducing changes in microenvironments like variations in cancer-associated fibroblasts, regulatory T cells (T_reg_ cells), and inflammatory mediators [46]. The other up-regulated miRNA is miR-4449, located on chromosome 4 with 22 predicted targets in miRDB (see Additional file 1: Table A2). Among the predicted targets, the cyclin-dependent kinase 5 regulatory subunit 2, p39 (CDK5R2) is detected as the most highly scored one. Previously, P39 had been introduced as a potential clinical prognostic marker for HCC [47]. Also, it had been detected that miR-4449 was a potential blood-based marker in multiple myeloma [48]. The present study suggested that miR-3158-5p and miR-4449 had significantly up-regulated in HCC patients with LT rejection. Even in analyzing sensitivity and specificity, these two miRNAs had AUC greater than 0.8 (Table 4). Therefore, they were proposed as a biomarker candidate for rejection in patients with HCC.

The third up-regulated miRNA in HCC patients with LT rejection was miR-4633-5p, located on chromosome 5 with more than 100 predicted targets in miRDB (see Additional file 1: Table A3). In 2015, a research group had further noted that miR-4633-5p could be significantly expressed in human epidermal growth factor receptor 2 (HER2)-positive breast carcinomas among 85 tested miRNAs versus normal population [49]. The last up-regulated studied miRNA in LT rejected patients with HCC is miR-4511, located on chromosome 15 with more than 400 predicted targets in miRDB (see Additional file 1: Table 4A). There are also few studies on the role of miRNA in LT and further studies are warranted to detect the exact role of this miR and its targets. The results of the present study showed the up-regulation of these two miRNAs in HCC patients with rejected LT.

It is of note that more than 97% of liver biopsies are hepatocytes and biliary cells, and the rest are Kupffer cells and endothelial ones. In LT rejected biopsies, some macrophages, plasma cells, and lymphocytes are also added to their population [50]. Given the fact that the selected miRNAs are expressed in the non-rejected group of patients and the majority of cells in biopsies are hepatocytes. Alterations in the expression profile of the selected miRNAs might be due to the rejection process in hepatocytes.

Putting together, noticing the fact that there is un urgent need for detecting biomarkers in diagnosis and prognosis of HCC, the findings of this study might be the first step in this pathway and seems to be preliminary keys for further confirmation of their regulatory role in rejection in LT patients with HCC. For instance, among down-regulated miRNAs, miR194-5p and miR548as-3p, both showed significant difference between rejected and nonrejected samples and the ROC curve analysis of these two miRNAs showed significant high specificity and sensitivity. Also, among up-regulated miRNAs, miR-4449 and miR-3158-5p can be considered to have important roles in causing rejection in such patients. Besides the ROC curve analysis of these two microRNAs showed significant high specificity and sensitivity. Further studies on detecting the important targets of these miRNAs, obviously would be a large finding in this field.

Although finding miRNAs as biomarkers might be very helpful for more accurate prognosis and diagnosis of HCC. This study was suffered from some limitations including: small sample size based on nature and prevalence of HCC, difficulties for follow up of patients and ethical issue related to collecting biopsy samples from normal tissue.

## Conclusion

In summary, the identification of miRNAs and small RNA species seems to represent only the tip of the iceberg. In addition, the prediction of an individual miRNA, its target, and even its function is still one of the biggest challenges in research. Therefore, based on these results, two down-regulated (miR194-5p and miR584as-3p) and two up-regulated (namely, miR-4449 and miR-3158-5p) miRNAs were introduced as candidate biomarkers for LT rejection in HCC patients which need to be confirmed by further studies. Even though the biological function of miRNAs and results of this research encourage researches to study more this subject.

## Supplementary information

**Additional file 1**. Extra plots and data of evaluated microRNA targets.

## Abbreviations

ALT: alanine transaminase
AST: aspartate transaminase
AFT: α-fetoprotein
HCC: hepatocellular carcinoma
miRNAs: microRNAs
3′-UTRs: 3′-untranslated regions
LNA-array: locked nucleic acid-array
FFPE: formalin-fixed paraffin-embedded
TEs: transposable elements
TGF-β: transforming growth factor β
DLBL: large B-cell lymphoma
T_reg_ cells: regulatory T cells
CRC: colorectal cancer
CDK5R2: cyclin-dependent kinase 5 regulatory subunit 2, p39
HER2: human epidermal growth factor receptor 2

## Availability of data and materials

The datasets used and/or analyzed during the current study are available from the corresponding author on reasonable request.
