# Supplementary material for: Alterations in MicroRNA gene expression profile in liver transplant patients with hepatocellular carcinoma
Source: BMC Gastroenterol. 2021 Jun 12;21:262. doi: 10.1186/s12876-020-01596-2 (PMC8199419; doi:10.1186/s12876-020-01596-2)
Supplement: Supplementary file 1 — Additional file 1. Extra plots and data of evaluated microRNA targets. [file 12876_2020_1596_MOESM1_ESM.docx]

Additional file

**MicroRNA Gene Expression Alterations in Hepatocellular Carcinoma Liver Transplanted Patients**


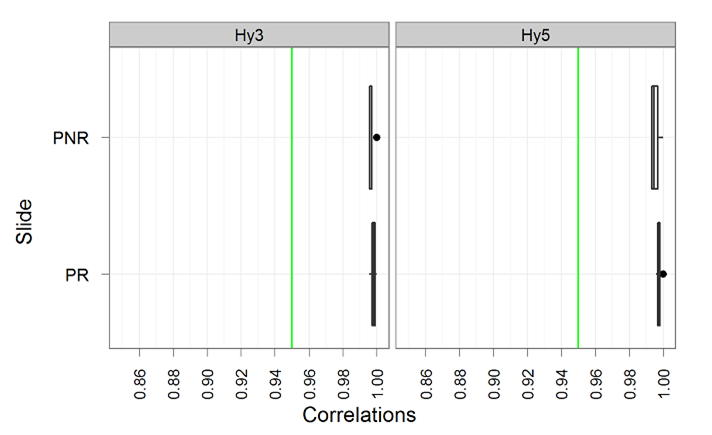


**Fig A1:** Box plot showing the correlation between spike-in controls on each slide to spike-in controls on the other slides in the study. A median (vertical black line) correlation higher than 0.95 (indicated by the green line) is considered as acceptable. Narrow boxes mean that spike-in controls on the given slide correlate similarly to most other slides. Black dots refer to outlier correlations deviating significantly from the slide’s average correlation to other slide's spike-in controls.

**
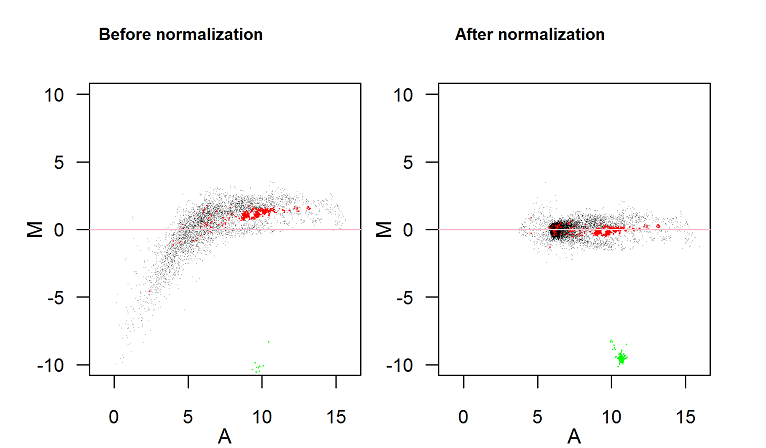
**

**Fig A2:** An example of an MA plot. All MA plots are found in the folder named MA plots. Green: Hy3 controls, red: spike-ins, black: all other probes. After normalization, the spots symmetrically scattered around the horizontal line M=0. The difference between the two channels (M) is now independent of the average intensity level of the two channels (A).

**Table-A1:** The table shows upper 95%target score predicted targets for hsa-miR-3158-5p in miRDB.

| **Gene Description** | **Gene Symbol** | **miRNA Name** | **Target**  **Score** | **Target**  **Rank** |
| --- | --- | --- | --- | --- |
| heat shock 70kda protein 12A | HSPA12A | hsa-miR-3158-5p | 99 | 1 |
| C-type lectin domain family 14, member A | CELEC14A | hsa-miR-3158-5p | 98 | 2 |
| KIAA0709 | KIAA0709 | hsa-miR-3158-5p | 98 | 3 |
| Secreted frizzled-related protein 1 | SFRP1 | hsa-miR-3158-5p | 98 | 4 |
| Inositol hexakisphosphate kinase 3 | IP6K3 | hsa-miR-3158-5p | 98 | 5 |
| fidgetin | FIGN | hsa-miR-3158-5p | 98 | 6 |
| Transforming growth factor, beta 2 | TGFB2 | hsa-miR-3158-5p | 98 | 7 |
| IKAROS family zinc finger 3 (Aiolos) | IKZF3 | hsa-miR-3158-5p | 98 | 8 |
| Fas associated factor family member 2 | FAF2 | hsa-miR-3158-5p | 97 | 9 |
| Angiomotin like 1 | AMOTL1 | hsa-miR-3158-5p | 97 | 10 |
| claspin | CLSPN | hsa-miR-3158-5p | 96 | 11 |
| BTAF1 RNA polymerase II, B-TAFIID transcription factor-associated, 170kDa | BATF1 | hsa-miR-3158-5p | 96 | 12 |
| KIAA1045 | KIAA1045 | hsa-miR-3158-5p | 96 | 13 |
| homeodomain interacting protein kinase 1 | HIPK1 | hsa-miR-3158-5p | 96 | 14 |
| Nuclear fragile X mental retardation protein interacting protein 2 | NUFIP2 | hsa-miR-3158-5p | 95 | 15 |
| zinc finger and BTB domain containing 44 | ZBTB44 | hsa-miR-3158-5p | 95 | 16 |
| Patched domain containing 1 | PTCHD1 | hsa-miR-3158-5p | 95 | 17 |
| E2F transcription factor 7 | E2F7 | hsa-miR-3158-5p | 95 | 18 |
| huntingtin interacting protein 1 related | HIP1R | hsa-miR-3158-5p | 95 | 19 |
| Adenosylhomocysteinase-like 1 | AHCL1 | hsa-miR-3158-5p | 95 | 20 |
| Myosin binding protein C, slow type | MYBPC1 | hsa-miR-3158-5p | 95 | 21 |
| RAS protein activator like 2 | RASAL2 | hsa-miR-3158-5p | 95 | 22 |

**Table-A2:** The table shows predicted targets for hsa-miR-4449 in miRDB.

| **Gene Description** | **Gene Symbol** | **miRNA Name** | **Target**  **Score** | **Target**  **Rank** |
| --- | --- | --- | --- | --- |
| cyclin-dependent kinase 5, regulatory subunit 2 (p39) | CDK5R2 | hsa-miR-4449 | 93 | 1 |
| hypermethylated in cancer 1 | HIC1 | hsa-miR-4449 | 87 | 2 |
| translocase of outer mitochondrial membrane 40 homolog (yeast) | TOMM40 | hsa-miR-4449 | 78 | 3 |
| diffuse panbronchiolitis critical region 1 | DPCR1 | hsa-miR-4449 | 77 | 4 |
| RNA binding motif protein 4 | RBM4 | hsa-miR-4449 | 76 | 5 |
| zinc finger homeobox 3 | ZFHX3 | hsa-miR-4449 | 70 | 6 |
| inducible T-cell co-stimulator | ICOS | hsa-miR-4449 | 69 | 7 |
| ribosomal protein L10 | RPL10 | hsa-miR-4449 | 69 | 8 |
| phosphatidylinositol- 3,4, 5-triphosphate-dependent Rac exchange factor 1 | PREX1 | hsa-miR-4449 | 68 | 9 |
| leucin repeat rich and fibronectin type III domain containing 1 | LRFN1 | hsa-miR-4449 | 68 | 10 |
| interleukin 7 receptor | IL7R | hsa-miR-4449 | 68 | 11 |
| chromosome 12 open reading frame 61 | C12orf61 | hsa-miR-4449 | 60 | 12 |
| serine/argenine repetitive matrix protin 2-like | LOC101928558 | hsa-miR-4449 | 59 | 13 |
| killin, p53-regulatd DNA replication inhibitor | KLIN | hsa-miR-4449 | 59 | 14 |
| CDK5 and ABL1 enzyme substrate 2-like | LOC101928038 | hsa-miR-4449 | 59 | 15 |
| homeobox B9 | HOXB9 | hsa-miR-4449 | 54 | 16 |
| armadillo repeat gene deleted in velocardiofacial syndrome | ARVCF | hsa-miR-4449 | 54 | 17 |
| solute carrier family 22, member 20 | SLC22A20 | hsa-miR-4449 | 54 | 18 |
| mitogen-activated protein kinase 11 | MAPK11 | hsa-miR-4449 | 54 | 19 |
| surfeit 6 | SURF6 | hsa-miR-4449 | 51 | 20 |
| suppressor of cytokine signaling 3 | SOCS3 | hsa-miR-4449 | 51 | 21 |
| STAR-related lipid transfer (START) domain containing 13 | STARD13 | hsa-miR-4449 | 50 | 22 |

**Table-A3:** The table shows upper 85% target score predicted targets for hsa-miR-4633-5p in miRDB.

| **Gene Description** | **Gene Symbol** | **miRNA Name** | **Target**  **Score** | **Target**  **Rank** |
| --- | --- | --- | --- | --- |
| excision repair cross-complementation group 6-like | ERCC6L | hsa-miR-44633-5p | 96 | 1 |
| solute carrier family 38, member 2 | SLC38A2 | hsa-miR-44633-5p | 96 | 2 |
| phosphatidylinositol transfer protein, cytoplasmic 1 | PITPNC1 | hsa-miR-44633-5p | 95 | 3 |
| fibronectin leucin rich transmembrane protein 3 | FLRT3 | hsa-miR-44633-5p | 95 | 4 |
| Ras association (RalGDS/AF-6) domain family member 5 | RASSF5 | hsa-miR-44633-5p | 95 | 5 |
| RAB11 family interacting protein 2 (class I) | RAB11FIP2 | hsa-miR-44633-5p | 94 | 6 |
| leptin | LEP | hsa-miR-44633-5p | 92 | 7 |
| basic helix-loop-helix family, member e41 | BHLHE41 | hsa-miR-44633-5p | 92 | 8 |
| G1 to S phase transition 1 | GSPT1 | hsa-miR-44633-5p | 91 | 9 |
| myocyte enhancer factor 2C | MEF2C | hsa-miR-44633-5p | 90 | 10 |
| NGFI-A binding protein 1 (EGR1 binding protein 1) | NAB1 | hsa-miR-44633-5p | 88 | 11 |
| adenylosuccinate synthase | ADSS | hsa-miR-44633-5p | 88 | 12 |
| neuronal cell adhesion molecule | NRCAM | hsa-miR-44633-5p | 88 | 13 |
| PDS5, regulator of cohesion maintenance, homolog B (S. cerevisiae) | PDS5B | hsa-miR-44633-5p | 86 | 14 |
| ubiquitin carboxyl-terminal hydrolase L5 | UCHL5 | hsa-miR-44633-5p | 86 | 15 |
| cadherin 13 | CDH13 | hsa-miR-44633-5p | 85 | 16 |
| centrosomal protein 44kDa | CEP44 | hsa-miR-44633-5p | 85 | 17 |
| Ras protein-specific guanin nucleotide releasing-factor 2 | RASGRF2 | hsa-miR-44633-5p | 85 | 18 |

**Table-A4:** The table shows upper 96% target score predicted targets for hsa-miR-4511 in miRDB.

| **Gene Description** | **Gene Symbol** | **miRNA Name** | **Target**  **Score** | **Target**  **Rank** |
| --- | --- | --- | --- | --- |
| oxidation resistance 1 | OXR1 | hsa-miR-4511 | 100 | 1 |
| ubiquitin specific peptidase 25 | USP25 | hsa-miR-4511 | 100 | 2 |
| sel-1 suppressor of lin-12-like (C. elegans) | SEL1L | hsa-miR-4511 | 99 | 3 |
| retinoic acid induced 14 | RAI14 | hsa-miR-4511 | 99 | 4 |
| male-specific lethal 2 homolog (Drosophila) | MSL2 | hsa-miR-4511 | 99 | 5 |
| chromosome 4 open reading frame 32 | C4orf32 | hsa-miR-4511 | 99 | 6 |
| early endosome antigen 1 | EEA1 | hsa-miR-4511 | 99 | 7 |
| RAP2C, member of RAS oncogene family | RAP2C | hsa-miR-4511 | 99 | 8 |
| calcyphosine 2 | CAPS2 | hsa-miR-4511 | 99 | 9 |
| N(alpha)-acetyltransferase 30, NatC catalytic subunit | NAA30 | hsa-miR-4511 | 99 | 10 |
| Sp3 transcription factor | SP3 | hsa-miR-4511 | 99 | 11 |
| cadherin 11, type 2, OB-cadherin (osteoblast) | CDH11 | hsa-miR-4511 | 98 | 12 |
| fragile X mental retardation, autosomal homolog 1 | FXR1 | hsa-miR-4511 | 98 | 13 |
| OTU deubiquitinase 4 | OTUD4 | hsa-miR-4511 | 98 | 14 |
| sterile alpha motif domain containing 4B | SAMD4B | hsa-miR-4511 | 98 | 15 |
| forkhead box J2 | FOXJ2 | hsa-miR-4511 | 98 | 16 |
| tyrosine 3-monooxygenase/tryptophan 5-monooxygenase activation protein, epsilon | YWHAE | hsa-miR-4511 | 97 | 17 |
| transmembrane protein 245 | TMEM245 | hsa-miR-4511 | 97 | 18 |
| small ubiquitin-like modifier 1 | SUMO1 | hsa-miR-4511 | 97 | 19 |
| RNA binding motif, single stranded interacting protein 1 | RBMS1 | hsa-miR-4511 | 97 | 20 |
| sterol carrier protein 2 | SCP2 | hsa-miR-4511 | 96 | 21 |
| transforming growth factor, beta receptor associated protein 1 | TGFBRAP1 | hsa-miR-4511 | 96 | 22 |
| fibronectin type III and SPRY domain containing 1-like | FSD1L | hsa-miR-4511 | 96 | 23 |
